# What is the best approach for patients with prolonged duration of untreated psychosis (DUP) - about 2 clinical cases with dup longer than 10 years

**DOI:** 10.1192/j.eurpsy.2021.1443

**Published:** 2021-08-13

**Authors:** R. Gomes, C. Santos, F. Moutinho

**Affiliations:** 1 Psychiatry And Mental Health Department, Hospital Garcia de Orta, E.P.E., Almada, Portugal, Portugal; 2 Psychiatry, Hospital Garcia de Orta, Almada, Portugal

**Keywords:** schizophrénia, duration of untreated psychosis, psychosis, clozapine

## Abstract

**Introduction:**

Studies have consistently found that many individuals with psychosis experience significant delays before receiving treatment. DUP refers to the period between the emergence of psychotic symptoms and the initiation of appropriate clinical treatment.

**Objectives:**

To review current knowledge on the best approach for patients with schizophrenia (SCZ) and prolonged DUP.

**Methods:**

Non-systematic review of literature through search on PubMed database, following the terms “DUP and treatment” and “impact of longer DUP”. Two clinical cases are described.

**Results:**

The clinical cases describe patients with SCZ with DUPs older than 10 years, in whom we could not achieve complete clinical remission after several therapeutic trials and whose prognosis was admitted as reserved. Longer DUP is an independent predictor of poorer outcome in SCZ, including the poor response to treatment and difficulty in achieving remission, predicting treatment resistance. Identifying treatment-resistant patients is crucial due to the importance of initiating clozapine as early as possible since the chances of responding are higher.

**Conclusions:**

DUP is a key prognostic variable in psychosis, revealing the significance of early treatment. Patients with long DUP should be regarded as at high risk of poor recovery. The detection of these patients enables clinicians to avoid unnecessary exposure to ineffective treatments while effective interventions are delayed. However, in view of adverse side effects of clozapine, future studies need to examine relevant predictors to detect accurately non-responders. We also suggest further studies to understand if there is correspondence between DUP and different stages of the disease that justify these results.

